# A Shallow Angle Short-Axis Out-of-Plane Approach Reduces the Rate of Posterior Wall Injuries in Central Venous Catheterization: A Simulation Study

**DOI:** 10.1155/2018/4793174

**Published:** 2018-09-10

**Authors:** Kunitaro Watanabe, Joho Tokumine, Alan Kawarai Lefor, Akira Motoyasu, Kumi Moriyama, Tomoko Yorozu

**Affiliations:** ^1^Department of Anesthesiology, Kyorin University School of Medicine, Tokyo, Japan; ^2^Department of Surgery, Jichi Medical University, Tochigi-ken, Japan

## Abstract

The short-axis out-of-plane approach (SAX-OOP) is commonly used in ultrasound-guided internal jugular vein catheterization. However, this approach has a risk of posterior vein wall injuries. The authors hypothesized that a shallow angle of approach may reduce the rate of posterior wall injuries compared with the conventional steep angle approach. The present study aimed to evaluate whether a difference in the angle of approach of the needle affects the rate of posterior wall injuries. The present study was a randomized crossover-controlled trial involving 40 medical residents, conducted in the clinical training center at a hospital with a residency program. The primary outcome measure was the rate of posterior vessel wall injuries. Subjects received a didactic lecture during which the instructors taught three SAX-OOP techniques including the conventional free-hand method (procedure C), a needle navigation system (procedure N), and a shallow puncture angle using a guidance system (procedure S). Participants were trained in these approaches under supervision and each technique tested in a simulation environment. Thirty-four of 40 residents had no previous experience with central venous catheterization and were included in the final analysis. The rate of posterior vessel wall injuries in procedure S (9%) was significantly lower than using the other approaches (procedure C, 53%; procedure N, 41%). In conclusion, a shallow angle of approach using the SAX-OOP technique resulted in significantly fewer posterior vein wall injuries in central venous catheterization compared with steep angle techniques.

## 1. Introduction

Ultrasound-guided internal jugular vein catheterization reduces the rate of complications compared with the anatomical landmark technique, although complications still occur in approximately 4% of procedures [1]. The most commonly used technique for ultrasound-guided internal jugular vein catheterization is the short-axis out-of-plane (SAX-OOP) approach [2]. This approach, however, occasionally results in posterior vein wall injury, which has a considerable risk of inadvertent arterial injuries [3]. The long-axis in-plane (LAX-IN) approach is generally associated with fewer posterior wall injuries [4, 5]. Innovations in technology may enable us to reduce the risk for posterior wall injuries using the SAX-OOP approach. A recent needle navigation system used with ultrasound imaging enables the operator to navigate the needle trajectory and depth in real time. Auyong et al. [6] reported that this needle navigation technology yielded a high success rate and reduced the rate of posterior wall injuries.

We conducted a pilot study that did not result in any improvement in the rate of posterior wall injuries using this needle navigation system but did have a shorter procedure duration (Table 1). Seven residents performed ultrasound-guided central vein venipuncture in a simulation environment, with and without the guidance system. Posterior wall injuries occurred in 71% of procedures using the navigation system and 71% using the free-hand technique. These results are different from those reported in the literature [6]. Further evaluation identified that the difference between our pilot study and previous results revealed that the authors used a shallow angle of approach for the needle. The pilot study we conducted used a steep angle of approach, as described in the guidelines of the American Society of Echocardiography (ASE) & Society of Cardiovascular Anesthesiologists (SCA) [2]. Therefore, the present study aimed to evaluate whether a difference in the angle of approach of the needle affects the rate of posterior wall injury.

## 2. Materials and Methods

The present study was reviewed and approved by the local Ethics Committee of Kyorin University [Tokyo, Japan]; Reception. No. 682-01) and registered in the University Hospital Medical Information Network Center Clinical Trials Registration System (UMIN000024559, Kunitaro Watanabe, 2016/10/25). Informed written consent was obtained from all participants. Subjects with previous experience performing central venous catheterization were excluded.

The study was performed using a commercially available ultrasound device (eZono4000®, eZono AG, Jena, Germany), equipped with novel, real time, and needle navigation technology [6]. This system enables free-hand navigation to visualize the needle trajectory and needle tip position using ultrasound imaging. The needle used was a 20-gauge (20G) metal introducer needle (effective length 32 mm, CV Legaforce EX®, Terumo Co., Tokyo, Japan) [7]. The simulator used was developed for ultrasound-guided internal jugular vein catheterization (UGP GEL®, Alfabio Co., Japan). The simulated internal jugular vein and carotid artery were located at 11 mm and 22 mm from the surface. The simulated internal jugular vein was connected to a water server tank through a tube to maintain pressure at 10 cmH_2_O, which was monitored by a pressure transducer.

Forty residents were initially enrolled in the study. Study participants received a didactic lecture to explain ultrasound-guided central venous catheterization. The instructors demonstrated how to perform three different approaches including the conventional free-hand SAX-OOP (procedure C) approach, the conventional SAX-OOP using the novel needle navigation system (procedure N), and the SAX-OOP technique with a shallow angle of approach and the needle navigation system (procedure S), using a simulator.

**Figure 1:**
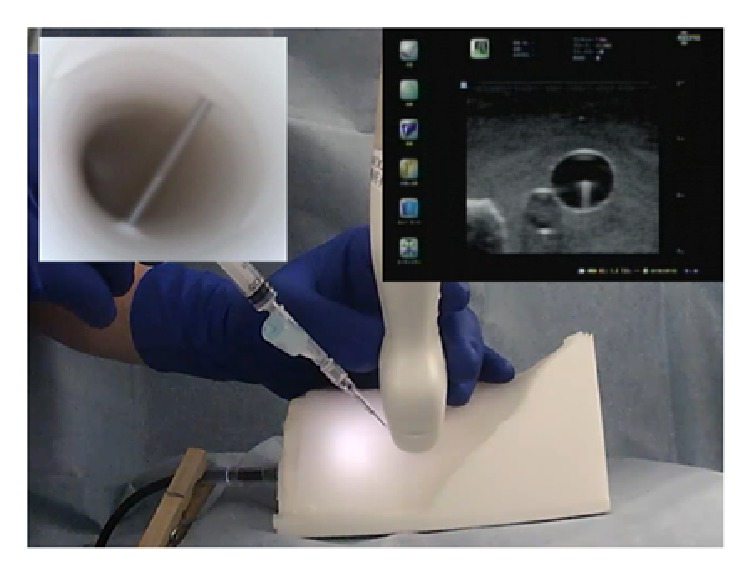
Conventional short-axis out-of-plane approach. The needle insertion site is close to the ultrasound probe, and the angle is adjusted to match the (simulated) internal jugular vein. This standard technique may result in a steep angle of the needle to match the internal jugular vein. Using this approach, posterior vein wall injuries were more common and difficult to identify.

**Figure 2:**
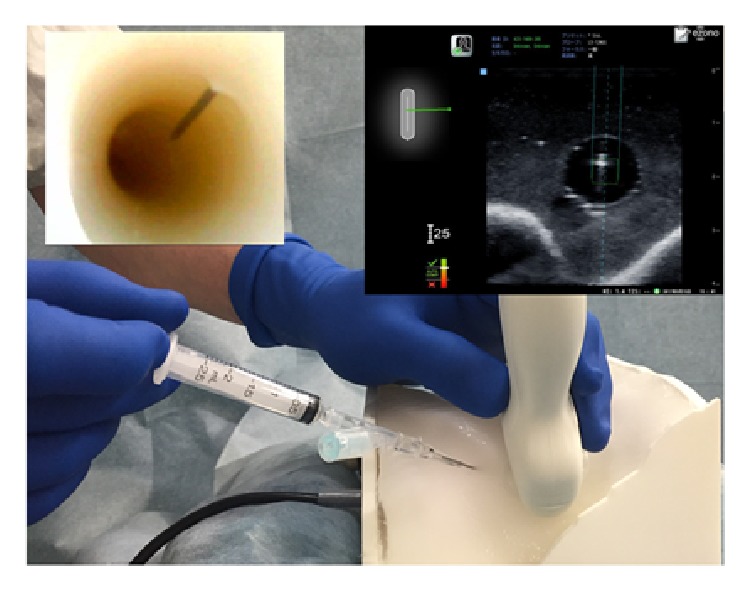
Short-axis out-of-plane approach with shallow angle of entry using a needle navigation system. The needle enters the skin further from the probe, approximately 1 cm cephalad, which allows for a shallow angle of entry toward the simulated internal jugular vein. The needle is inclined at approximately 30° to the target vein.

Guidelines from the ASE and the SCA recommend that the needle enters the skin close to the ultrasound probe. The angle is then adjusted to match the target internal jugular vein [2]. Following this standard technique may result in a steep angle between the needle and the internal jugular vein (Figure 1). Instructors taught the standard steep angle SAX-OOP method for procedure C and the SAX-OOP using the needle navigation system, with the entry site close to the probe, for procedure N. For procedure S, the needle entry site into the skin was further from the probe—approximately 1 cm cephalad—resulting in a shallow angle of entry to the target vein. The needle was inclined at approximately 30° to the skin in procedure S (Figure 2).

During simulation training, participants could observe the inside of the simulated vessel using an endoscope, which is part of the simulator, and could confirm whether the needle tip was inside or outside of the vessel. Simulation training continued until participants were confident in their ability with the technique and were given advice by the instructor as desired. The training did not exceed 2 h for any participant.

After simulation training, a test was conducted using each approach. All participants performed all three approaches in the test. The endoscopic view inside of the simulated vessel was no longer visible to the participants but was recorded during the test. The video recordings were sequentially numbered. The video numbers were later randomized by computer to assure anonymity. The technique used and individual identification were concealed for the evaluation. Two senior physicians, who did not participate in the test, observed the recorded videos and evaluated whether the procedure was performed successfully and whether a posterior wall injury occurred.

This was a randomized crossover-controlled trial. The sequence for each participant to perform each technique was randomly decided using a random number table. The primary outcome measure was the rate of posterior vessel wall injuries. Secondary outcomes include success rate, needle passes until success, procedure duration, and unanticipated arterial injury. A questionnaire was completed by participants to assess their comfort with the procedures using a 5-point Likert scale (5 = very comfortable, 1 = not at all comfortable) and their preferences in clinical practice.

## 3. Statistical Analysis

There are no studies which compare different puncture angles. The method described by Auyong et al. used a slightly shallower insertion angle compared to the ASE and SCA recommended technique [6]. We performed a power analysis to determine an order of magnitude result. The study reported by Auyong et al. found the incidence of posterior vessel wall puncture without and with needle navigation to be 49% and 13%, respectively [6]. The sample size required for 80% power at *ɑ* = 0.05 was estimated to be 31 participants. In this study, we included 40 residents to guard against drop-outs and exclusions.

Fisher's exact test was used to evaluate the rate of posterior wall puncture, rate of arterial puncture, success rate, and Likert scale scores. Analysis of variance and Bonferroni correction were used to compare continuous variables. Numerical values were expressed as percentages (%) or as mean ± standard deviation for normally distributed variables and median (interquartile range) for nonnormally distributed variables. A p value < 0.05 was considered to be statistically significant. Statistical analyses were performed using EZR using R commander, version 1.32 (Saitama Medical Center, Jichi Medical University, Saitama, Japan) [8].

## 4. Results

Thirty-four residents were enrolled in the study (six of the 40 originally recruited residents had previously performed central venous catheterization and were, therefore, excluded). A summary of outcome measures and resident preferences is presented in Table 2. The rate of posterior vessel wall puncture for procedure S (9%) was significantly lower than the other two procedures (p < 0.01). There was no statistically significant difference between procedures C (53%) and N (41%).

There was a statistically significant difference in procedure duration among the three procedures (p = 0.02). The duration for procedure N (43 ± 15 s) was shorter than for procedure C (56 ± 21 s) (p = 0.01). The clinical relevance of this difference (<10s) is marginal. There was no difference between procedures S (49 ± 18) and C. There was no difference in the number of needle passes among the three procedures (p = 0.37). There were no arterial injuries in any of the three procedures, and all had a 100% success rate.

Comfort with performing procedure N was significantly higher than for procedure C (p = 0.02). There was no significant difference in comfort with the procedure between procedures S and C (p = 0.1) or procedures N and S (p = 1.0). The preferred procedure in clinical practice assessed by the survey after the study was significantly the highest for the shallow angle SAX-OOP (procedure S) approach (56%).

## 5. Discussion

The conventional SAX-OOP approach has a risk of posterior vein wall injury. The present study shows that this risk is not due to the SAX-OOP approach itself, but rather to the steep angle of needle insertion. If the angle of approach is decreased from 60° to 30°, the calculated needle trajectory path from the anterior wall to the posterior wall is approximately 1.7 times longer. This may explain why a shallow angle reduces the rate of posterior wall injury. The reduced rate of posterior wall injuries may also be related to needle handling skill during ultrasound guidance. Experts performing ultrasound-guided central venous catheterization may stop advancing the needle when the needle tip enters the ultrasound beam, while novice operators usually do not stop at that point. Experts do not sense a risk using a steep angle of approach and have adequate skill handling the needle. The conventional steep angle of insertion has the benefit of easily advancing the needle directly to the target vein because the needle entry site into the skin is close to the ultrasound beam. However, the conventional steep angle used in the SAX-OOP approach has the drawback of a higher rate of posterior wall injuries.

Both the LAX-IN and oblique approaches usually require a shallow angle of approach, which may partially explain why these approaches are not usually associated with posterior wall injuries [9–11]. The LAX-IN approach is difficult to use for internal jugular vein catheterization due to limitations related to manipulating the ultrasound probe on the neck. A small-footprint probe is required to use the LAX-IN approach in smaller adult patients. Performing the LAX-IN approach also requires precise scanning skills to place the probe on the longitudinal center-line to prevent anterior wall to lateral wall injuries [12]. Advancing the needle within the ultrasound beam requires skilled hands. The medial oblique approach is an alternative option to prevent posterior wall injuries. This also requires a highly experienced operator to advance the needle within the ultrasound beam [13].

As mentioned, the needle trajectory path from the anterior to the posterior wall will be longer when the angle of approach is shallower, which follows from simple geometric considerations. This simple calculation may be affected by dilation of the internal jugular vein with patients in the Trendelenburg position and/or during a Valsalva maneuver, or constriction of the vein with dehydration and/or decreased circulating volume. Needle size may also be a factor because a smaller-gauge needle can penetrate the anterior vein wall more easily than a larger needle and may be helpful to prevent posterior vein wall injuries. We recommend using a <20G needle to reduce the rate of posterior wall injuries.

A shallow angle of approach of the needle reduces the rate of posterior wall injuries and requires the skin entry site to be further from the ultrasound probe. However, this increased distance may be associated with risk. Operators can use a shallow angle of insertion while still using the conventional steep angle approach. This technique is simple: the operator begins with the conventional approach and alters the angle from steep to shallow when the needle tip reaches the anterior vein wall.

## 6. Conclusions

This study demonstrates that the increased number of posterior vein wall injuries associated with the SAX-OOP approach is due to the relatively steep angle of approach. A shallow angle of approach resulted in fewer posterior vein wall injuries. This procedural complication is not limited to the SAX-OOP technique. Further innovations to limit this complication are anticipated.

## Figures and Tables

**Table 1:** Results of a previous pilot study (unpublished).

Outcome	Navigation system	Free-hand technique	P value (Statistical test)
Posterior vessel wall injury (%)	71	71	1.00 (Fisher's exact test)
Arterial injury (%)	0	0	1.00 (T-test)
Overall success rate (%)	100	100	1.00 (Fisher's exact test)
Needle passes until success, mean ± SD	1.0 ± 0	1.1 ± 0.38	0.34 (T-test)
Procedure duration (s), mean ± SD	74 ± 46	145 ± 70	0.04 (T-test)

**Table 2:** Summary of outcome measures and resident preferences.

Outcome	Procedure C (Conventional)	Procedure N (Navigation)	Procedure S (Shallow angle + navigation)
Posterior vessel wall injury (%)	53	41	9
Arterial injury (%)	0	0	0
Overall success rate (%)	100	100	100
Needle passes until success, mean ± SD	1.0 ± 0	1.0 ± 0.2	1.0 ± 0
Procedure duration (s), mean ± SD	56 ± 21	43 ± 15	49 ± 18
Comfort with procedure*∗*, median (IQR)	4 (3–4)	4 (4–5)	4 (4–5)
Preferred procedure (%)	3	41	56

*∗*Scored according to a Likert scale (1-5 [5 is best]). IQR, interquartile range.

## Data Availability

Data are available in Supplementary Materials.

## Supplementary Materials

Supplementary Materials Supplementary files include raw data of this research, which are demographic data of the participants, data of outcomes in each procedure types, and answers to the postseminar questionnaire.
