# Supplementary material for: A Shallow Angle Short-Axis Out-of-Plane Approach Reduces the Rate of Posterior Wall Injuries in Central Venous Catheterization: A Simulation Study
Source: Biomed Res Int. 2018 Sep 10;2018:4793174. doi: 10.1155/2018/4793174 (PMC6151846; doi:10.1155/2018/4793174)
Supplement: Supplementary Materials — Supplementary files include raw data of this research, which are demographic data of the participants, data of outcomes in each procedure types, and answers to the postseminar questionnaire. [file 4793174.f1.pdf]

Demographic data of participants

Participants No.	Age (year)	Medical working experience（months）	Sex (M/F)	Experience of CVC
1	28	10	M	NO
2	28	10	M	NO
3	28	11	M	NO
4	26	10	M	NO
5	30	11	M	NO
11	28	12	M	NO
12	27	1	M	NO
13	28	1	F	NO
14	26	1	M	NO
15	25	1	F	NO
16	28	2	F	NO
17	35	3	M	NO
18	26	2	M	NO
19	29	2	M	NO
21	24	2	F	NO
22	25	3	F	NO
23	24	3	F	NO
24	25	3	M	NO
25	25	4	M	NO
26	35	4	M	NO
27	26	4	F	NO
28	24	4	M	NO
29	26	4	F	NO
30	24	4	F	NO
31	25	4	M	NO
32	26	4	M	NO
33	24	5	M	NO
34	25	5	F	NO
35	24	5	F	NO
36	29	4	M	NO
37	26	5	M	NO
38	29	5	M	NO
39	25	5	F	NO
40	25	5	M	NO

Results (outcomes)

Participant No.	Procedure type	Posterior vessel wall injury	Arterial injury	Needle Passes (n)	Procedure duration (sec)
1	C	Injury	NO	1	70
2	C	Injury	NO	1	25
3	C	No injury	NO	1	44
4	C	Injury	NO	1	32
5	C	No injury	NO	1	42
11	C	No injury	NO	1	25
12	C	No injury	NO	1	78
13	C	No injury	NO	1	28
14	C	Injury	NO	1	44
15	C	No injury	NO	1	58
16	C	Injury	NO	1	50
17	C	Injury	NO	1	128
18	C	Injury	NO	1	56
19	C	Injury	NO	1	65
21	C	No injury	NO	1	59
22	C	Injury	NO	1	52
23	C	No injury	NO	1	52
24	C	Injury	NO	1	39
25	C	No injury	NO	1	77
26	C	No injury	NO	1	61
27	C	No injury	NO	1	42
28	C	Injury	NO	1	43
29	C	Injury	NO	1	41
30	C	Injury	NO	1	42
31	C	Injury	NO	1	93
32	C	Injury	NO	1	85
33	C	Injury	NO	1	48
34	C	Injury	NO	1	65
35	C	No injury	NO	1	63
36	C	No injury	NO	1	66
37	C	No injury	NO	1	67
38	C	No injury	NO	1	76
39	C	No injury	NO	1	38
40	C	Injury	NO	1	58
1	N	Injury	NO	1	35
2	N	Injury	NO	1	31
3	N	Injury	NO	2	26
4	N	No injury	NO	1	40
5	N	No injury	NO	1	31
11	N	No injury	NO	1	19
12	N	Injury	NO	1	41
13	N	No injury	NO	1	30
14	N	No injury	NO	1	26
15	N	No injury	NO	1	65
16	N	No injury	NO	1	49
17	N	Injury	NO	1	35
18	N	Injury	NO	1	36
19	N	No injury	NO	1	46
21	N	Injury	NO	1	71
22	N	Injury	NO	1	85
23	N	Injury	NO	1	42
24	N	Injury	NO	1	51
25	N	Injury	NO	1	52
26	N	No injury	NO	1	33
27	N	No injury	NO	1	35
28	N	Injury	NO	1	44
29	N	No injury	NO	1	43
30	N	No injury	NO	1	37
31	N	No injury	NO	1	47
32	N	No injury	NO	1	49
33	N	Injury	NO	1	41
34	N	Injury	NO	1	28
35	N	No injury	NO	1	38
36	N	No injury	NO	1	69
37	N	No injury	NO	1	46
38	N	No injury	NO	1	67
39	N	No injury	NO	1	29
40	N	No injury	NO	1	59
1	S	No injury	NO	1	39
2	S	No injury	NO	1	31
3	S	No injury	NO	1	27
4	S	No injury	NO	1	28
5	S	No injury	NO	1	35
11	S	No injury	NO	1	22
12	S	No injury	NO	1	50
13	S	No injury	NO	1	30
14	S	No injury	NO	1	30
15	S	No injury	NO	1	41
16	S	No injury	NO	1	43
17	S	Injury	NO	1	53
18	S	No injury	NO	1	45
19	S	No injury	NO	1	45
21	S	No injury	NO	1	73
22	S	Injury	NO	1	95
23	S	Injury	NO	1	69
24	S	No injury	NO	1	47
25	S	No injury	NO	1	41
26	S	No injury	NO	1	96
27	S	No injury	NO	1	50
28	S	No injury	NO	1	38
29	S	No injury	NO	1	52
30	S	No injury	NO	1	56
31	S	No injury	NO	1	64
32	S	No injury	NO	1	76
33	S	No injury	NO	1	36
34	S	No injury	NO	1	36
35	S	No injury	NO	1	43
36	S	No injury	NO	1	69
37	S	No injury	NO	1	69
38	S	No injury	NO	1	42
39	S	No injury	NO	1	40
40	S	No injury	NO	1	54

Results (Questionnaire)

Participants No.	Comfort grading against Procedure C	Comfort grading against Procedure N	Comfort grading against Procedure S	Choice of preferred procedure
1	4	3	5	Procedure S
2	4	2	5	Procedure S
3	3	2	5	Procedure S
4	3	3	5	Procedure S
5	2	5	4	Procedure N
11	4	5	4	Procedure N
12	3	5	5	Procedure S
13	3	4	5	Procedure S
14	4	5	4	Procedure N
15	4	5	3	Procedure N
16	2	4	4	Procedure S
17	2	3	4	Procedure S
18	4	4	4	Procedure S
19	4	4	5	Procedure S
21	5	4	4	Procecure C
22	4	5	5	Procedure N
23	5	4	3	Procedure S
24	4	5	4	Procedure N
25	4	5	3	Procedure N
26	4	5	2	Procedure N
27	4	4	2	Procedure N
28	2	5	4	Procedure N
29	4	5	4	Procedure S
30	3	4	3	Procedure N
31	3	4	4	Procedure N
32	3	4	5	Procedure S
33	3	4	5	Procedure S
34	3	4	4	Procedure S
35	3	4	4	Procedure S
36	4	5	4	Procedure N
37	3	4	5	Procedure S
38	4	5	3	Procedure S
39	4	4	4	Procedure S
40	5	5	2	Procedure N
